# Resection vs. Ligation vs. Preservation of the Thoracic Duct During Esophagectomy for Cancer: A Systematic Review and Meta-Analysis

**DOI:** 10.3390/cancers17060967

**Published:** 2025-03-13

**Authors:** David J. Nijssen, Dillen C. van der Aa, Mahsoem Ali, Geert Kazemier, Faridi S. Jamaludin, Wietse J. Eshuis, Mark I. van Berge Henegouwen, Suzanne S. Gisbertz

**Affiliations:** 1 Department of Surgery, Amsterdam UMC location University of Amsterdam, Meibergdreef 9, 1105 AZ Amsterdam, The Netherlands; 2 Cancer Center Amsterdam, Cancer Treatment and Quality of Life, 1066 CX Amsterdam, The Netherlands; 3 Department of Gastroenterology and Hepatology, Amsterdam Gastroenterology Endocrinology Metabolism, Amsterdam UMC location University of Amsterdam, Meibergdreef 9, 1105 AZ Amsterdam, The Netherlands; 4 Department of Surgery, Amsterdam UMC location Vrije Universiteit, De Boelelaan 1118, 1081 HV Amsterdam, The Netherlands; 5 Medical Library AMC, Amsterdam UMC location University of Amsterdam, Meibergdreef 9, 1105 AZ Amsterdam, The Netherlands

**Keywords:** esophagectomy, thoracic duct, resection, preservation, ligation, chyle leakage, morbidity

## Abstract

Background/Objectives: The effect of thoracic duct (TD) management—resection, ligation, or preservation—during esophagectomy for cancer remains controversial. This systematic review and meta-analysis aimed to assess the impact of TD management strategies on oncological outcomes and surgical morbidity. Methods: A systematic review and meta-analysis were performed following PRISMA reporting guidelines. Searches of OVID, Embase, and Web of Science identified studies comparing thoracic duct resection or TD ligation with TD preservation in esophagectomy for cancer. Outcomes included 5-year overall survival (OS), postoperative morbidity, postoperative chyle leakage, lymph node yield, and length of stay. Random-effects meta-analyses using the Hartung-Knapp-Sidik-Jonkman variance correction were conducted. Results: A total of 17 studies involving 4200 patients were included. TD resection was associated with a significantly higher lymph node yield (mean difference [MD]: 4, 95% CI: 0 to 8, *p* = 0.043), but also increased risk of chyle leakage (odds ratio [OR]: 2.41, 95% CI: 1.04–5.61, *p* = 0.044). There was no significant improvement in 5-year OS with TD resection compared to TD preservation (hazard ratio [HR]: 0.94, 95% CI: 0.76–1.17, *p* = 0.48). TD ligation showed no significant differences in 5-year OS (HR: 1.15, 95% CI: 0.81–1.63, *p* = 0.33) or morbidity compared to TD preservation. Certainty of evidence was low across outcomes. Conclusions: TD resection increases lymph node yield but is associated with higher rates of chyle leakage, without a significantly improved overall survival. TD ligation does not significantly affect oncological or surgical outcomes compared to TD preservation. A higher grade of evidence is needed to determine the definitive oncological and surgical impact of TD management strategies.

## Simple Summary

The thoracic duct is the body’s largest lymphatic vessel, responsible for transporting lymph fluid, which helps in immune function and nutrient absorption. During surgery for esophageal cancer, surgeons may remove, seal, or preserve this duct, but the best approach remains uncertain. This study analyzed data from 17 studies involving 4200 patients to evaluate how different management strategies affect recovery and long-term survival. Removing the thoracic duct led to a higher number of lymph nodes being examined. However, this approach also increased the risk of chyle leakage, a complication where lymphatic fluid leaks into the chest. Despite this, removing the thoracic duct did not improve the chances of surviving for five years. Sealing the thoracic duct instead of removing it did not impact survival or surgical complications. More research is needed to determine whether different management strategies for the thoracic duct can improve surgical and cancer-related outcomes.

## 1. Introduction

Radical esophagectomy combined with neoadjuvant or perioperative chemo (radio) therapy is the standard treatment with curative intent for resectable esophageal cancer [1]. An en bloc thoracic duct (TD) resection is usually integrated as part of the esophagectomy, as this anatomical compartment may contain lymph nodes or lymph node metastases [2]. The TD is the main lymphatic vessel, responsible for draining about three-quarters of the body’s lymph fluid. It facilitates the transport of ingested fats, drains lymph from the gastrointestinal vascular bed, and delivers it to the central veins in the neck [3]. Intrathoracically, the thoracic duct ascends through the posterior mediastinum, shifting from the right to the left of the esophagus as it approaches the neck, where it lies posterior and lateral to the left of the esophagus, though its exact position can vary among individuals [4]. Due to this close anatomical proximity to the esophagus, the TD is at risk for damage during esophagectomy [5]. Lymph fluid carried by the TD is referred to as chyle once it has absorbed ingested fats from the small intestines, making its content rich in chylomicrons and triglycerides. Iatrogenic injury to the TD can therefore cause chyle leakage, which can lead to complications like chylothorax if it accumulates in the pleural cavity. Chyle leakage remains a relatively common complication after esophageal surgery, with an approximate incidence reported between 1% and 21% after esophagectomy [6,7,8]. It is associated with a longer duration of thoracic drainage and hospital stay, independent of the presence of other complications. Furthermore, chyle leakage may also increase the risk of pulmonary complications and intensive care admission. These factors have a negative impact on the in-hospital costs and likely also on societal costs. Additionally, reports indicate that chyle leakage is independently associated with reduced overall survival [9].

It remains controversial whether the TD should be routinely resected, ligated, or preserved during radical esophagectomy for cancer [10], as evidence with regard to both morbidity and survival is conflicting. Certain high-volume oncological centers favor esophagectomy with routine en bloc resection of the TD because it is believed to improve the radicality of the mediastinal lymphadenectomy and thereby the oncological outcome [11]. However, resection of the TD may increase surgical morbidity rates, including chyle leakage, and even left recurrent laryngeal nerve palsy due to more radical lymphadenectomy [12]. Furthermore, it is unclear whether an increase in mediastinal lymph node yield translates to an improvement in overall or disease-free survival rates [13].

Emerging imaging techniques such as indocyanine green fluorescence lymphography may potentially aid a safe resection and ligation as well as preservation of the TD [14,15], reducing the incidence of chyle leakage in all groups. However, the question of whether resection, ligation, or preservation is better from an oncological standpoint still needs to be answered.

This study aims to systematically review and conduct a meta-analysis of the existing literature on TD resection, TD ligation, and TD preservation to assess their impact on morbidity and short- and long-term oncological outcomes, evaluate evidence quality, and identify gaps in current knowledge.

## 2. Material and Methods

This systematic review with meta-analysis was reported following the guidelines of the Preferred Reporting Items for Systematic Review and Meta-Analysis Protocols (PRISMA) statement [16]. The PRISMA checklist is available in Supplementary File S1. This study was registered in the International Prospective Register of Systematic Reviews (PROSPERO) database on 9 May 2023 with the registration number CRD42023425268. The literature search was performed using OVID, Embase, and Web of Science databases up to June 2024, with no restrictions on language or region. The search terms and MeSH headings used were (“esoph*”) AND (“thoracic duct”), combined with Boolean operators. The full search is available in Supplementary File S2.

After removing duplicates, titles and abstracts were initially screened to identify articles of interest using Rayyan [17]. These articles then underwent full-text analysis and data extraction. Additionally, reference lists of retrieved articles were manually searched for further relevant references. The study selection was conducted independently by two researchers (D.C.v.d.A. and D.J.N.), with any discrepancies resolved through co-authors review and consensus. Two reviewers independently evaluated the trials for risk of bias using the methodology outlined in version 2 of the Cochrane Library Handbook (RoB2 + ROBINS-I) [18,19]. The certainty of evidence of each outcome domain was assessed using the GRADE approach (available in Supplementary File S3).

### 2.1. Inclusion and Exclusion Criteria

Eligible studies included those comparing TD resection to TD preservation and TD ligation and TD preservation in radical esophagectomy for esophageal cancer in adults. The primary outcome was overall survival (OS). Secondary outcomes of interest were postoperative complications, the incidence of chyle leakage, postoperative severe morbidity (CD ≥ 3), lymph node yield (LNY) in the surgical specimen, and length of hospital stay (LOS). Review articles, case reports, conference abstracts, or studies not available in English were excluded.

### 2.2. Statistical Analysis

Meta-analyses were conducted using R, version 4.4.1 (R Foundation for Statistical Computing), if three or more studies reported on the same outcome of interest. When studies reported a mean and standard deviation, the effect measure was expressed as a mean difference. For studies using other descriptive modes, such as median and interquartile range, a mean difference was estimated. We used the method from Hozo et al. to estimate the mean and variance [20].

In accordance with the methods described by Tierney et al., we extracted summary time-to-event data from included studies and incorporated them into our meta-analysis [21]. For studies reporting time-to-event outcomes such as overall survival, we used the reported hazard ratios (HRs) where available. In cases where HRs were not provided, we reconstructed them using the available data according to the methods provided by Tierney et al. [21] (e.g., the number of events, total participants, and *p*-values).

Following the guidelines set by the Cochrane Statistical Methods Group, two improved meta-analysis models were applied. Pooled effect estimates were derived using a random-effects meta-analysis model, with a restricted maximum likelihood estimator and Jackson’s modification of the Hartung-Knapp-Sidik-Jonkman variance correction to control the Type I error rate. For binary endpoints, the meta-analysis model consisted of a Paule–Mandel estimator and Jackson’s modification of the Hartung-Knapp-Sidik-Jonkman variance correction. A *p*-value of less than 0.05 was considered statistically significant.

## 3. Results

### 3.1. Systematic Review

**Figure 1:**
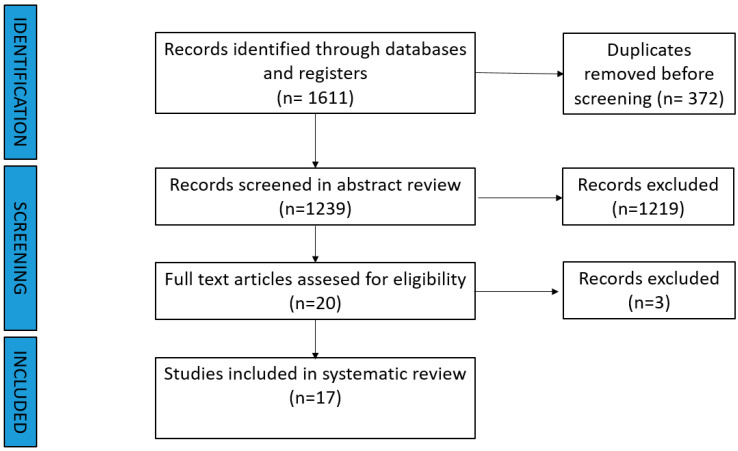
PRISMA.

The literature search initially identified 1611 studies. After removing duplicates, 17 studies remained for title and abstract review. The screening process is shown in Figure 1. Detailed results of the literature search and study selection process are provided in Supplementary File S2.

The ROBINS-I tool (Cochrane, London, UK) was applied to assess bias risk across all 17 studies (Table 1 and Table 2). Serious risk of bias was noted in the studies by Yoshida et al. [22], Matsuda et al. [23], Oshikiri et al. (2019) [13], Hou et al. [24], and Chen et al [25], largely due to inadequate control over key confounding factors such as neoadjuvant therapy and tumor staging.

The remaining studies were classified as having a moderate risk of bias. Although they used propensity score matching to address confounders, the lack of blinding during outcome assessment posed a limitation. Additionally, selective reporting may have been an issue, as results were reported across multiple patient subgroups with various stages of cancer and neoadjuvant treatment.

### 3.2. Data Review and Meta-Analysis: Resection vs. Preservation of the Thoracic Duct

#### 3.2.1. 5-Year Overall Survival

A total of five studies reported the 5-year OS after TD resection or (compared to?) TD preservation. Tanaka et al. (2021) [27] reported improved survival with TD resection (HR: 0.80, 95% CI: 0.69–0.94), while Oshikiri et al. (2023) [31], Oshikiri et al. (2022) [26], and Yoshida et al. (2019) [22] found no significant difference (HR: 0.95, 95% CI: 0.86–1.05; HR: 0.95, 95% CI: 0.64–1.41; HR: 0.91, 95% CI: 0.65–1.28) [22,26,27,31]. Oshikiri et al. (2019) demonstrated numerically more favorable survival after TD resection, though not statistically significant (HR: 1.39, 95% CI: 0.96–2.02) [13]. The pooled HR based on four studies was 0.94 (95% CI: 0.76–1.17, *p* = 0.48), with moderate heterogeneity (I^2^ = 53%, τ^2^ = 0.01).

#### 3.2.2. Chyle Leakage

**Figure 2:**
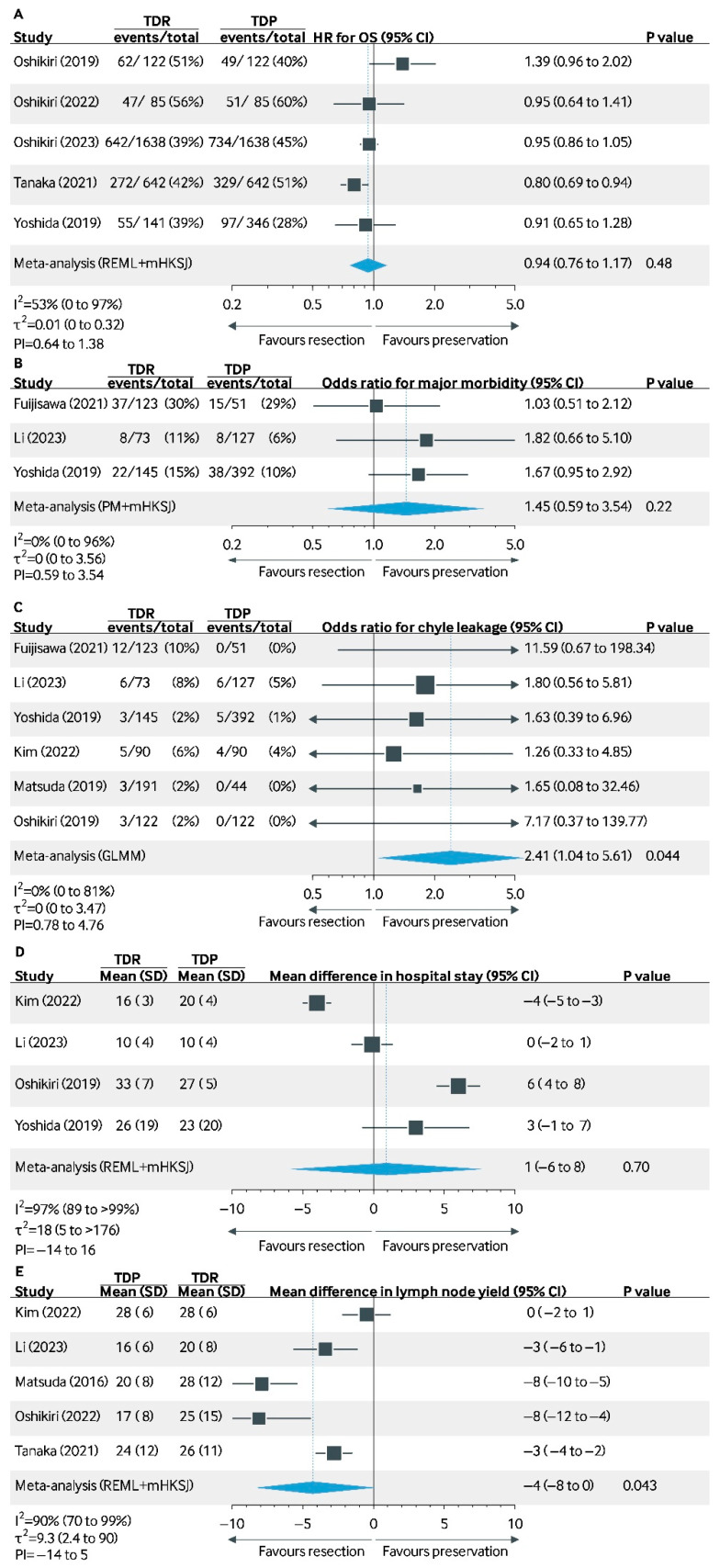
Meta-analysis of outcomes for thoracic duct resection vs. preservation [13,22,23,26,27,28,29,30,31,32]. Subfigures: (**A**): 5-year Overall Survival (OS); (**B**): Major Morbidity (Clavien–Dindo ≥ 3); (**C**): Incidence of Chyle Leakage; (**D**): Difference in Hospital Stay; (**E**): Difference in Lymph Node Yield. Abbreviations: TDR, thoracic duct resection; TDP, thoracic duct preservation; REML, restricted maximum likelihood; mHKSJ, Jackson’s modification of the Hartung-Knapp-Sidik-Jonkman variance correction; GLMM, generalized linear mixed model; PI, prediction interval; PM, Paule–Mandel.

The incidence of chyle leakage in the TD resection group ranged from 2% to 10%, whereas in the TD preservation group, this ranged from 0% to 5% (Figure 2). Fujisawa et al. (2021) reported a higher risk of chyle leakage in TD resection (OR: 11.59, 95% CI: 0.67–198.34), although not significant [28]. Kim et al. (2022) [29] and Li et al. (2023) [36] reported no significant differences (OR: 1.26, 95% CI: 0.33–4.85; OR: 1.80, 95% CI: 0.56–5.81, respectively) [29]. Yoshida (2019), Matsuda (2019), and Oshikiri (2019) also showed variable but non-significant results (OR: 1.63, 95% CI: 0.39–6.96; OR: 1.65, 95% CI: 0.08–32.46; OR: 7.17, 95% CI: 0.37–139.77, respectively) [13,22,32]. Overall, the pooled OR was 2.41 (95% CI: 1.04–5.61, *p* = 0.044), showing a significant increase in chyle leakage incidence with TD resection, with no evidence for heterogeneity (I^2^ = 0%, τ^2^ = 0).

#### 3.2.3. Incidence of Postoperative Complications

Matsuda et al. (2019) reported no significant differences in the incidence of anastomotic leakage (16% vs. 14.1%, *p* = 0.828), pneumonia (16% vs. 17.2%, *p* = 0.763), and recurrent laryngeal nerve (RLN) palsy (5% vs. 7.3%, *p* = 0.509) between TD preservation and TD resection [32]. Similarly, Fujisawa et al. (2021) found comparable rates of Clavien–Dindo (CD) grade III morbidity (29.4% vs. 30.1%, *p* = 0.930), anastomotic leakage (15.7% vs. 11.4%, *p* = 0.437), and pneumonia (19.6% vs. 23.6%, *p* = 0.691) [28]. Kim et al. (2022) also reported no statistically significant differences in rates of pneumonia (13.3% vs. 10.0%, *p* = 0.643), RLN palsy (26.7% vs. 25.6%, *p* = 1.000), or anastomotic leakage (20.0% vs. 12.2%, *p* = 0.223) [29]. Li et al. (2023) observed that pneumonia and RLN palsy rates remained comparable between the groups (11.0% vs. 11.0%, *p* = 0.242; 16.4% vs. 38.4%, *p* = 0.342), with no significant difference in anastomotic leakage (2.7% vs. 6.8%, *p* = 1.0) [30]. However, Yoshida et al. (2019) reported significant findings in certain areas, noting higher rates of pneumonia (14.5% vs. 8.4%, *p* = 0.038) and pulmonary morbidity (26.9% vs. 12.2%, *p* < 0.001) in the TD resection group, though anastomotic leakage was significantly lower in TD resection compared to TD preservation (8.3% vs. 14.8%, *p* = 0.046) [22].

#### 3.2.4. Major Morbidity (Clavien–Dindo ≥ 3)

TD resection vs. TD preservation was assessed for morbidity (CD ≥ 3). Fujisawa et al. (2021), Li et al. (2023), and Yoshida et al. (2019) found no significant difference in major morbidity between groups (OR: 1.03, 95% CI: 0.51–2.12; OR: 1.82, 95% CI: 0.66–5.10; OR: 1.67, 95% CI: 0.95–2.92) [22,28,30]. The overall pooled OR was 1.45 (95% CI: 0.59–3.54, *p* = 0.22), with no evidence for between-study heterogeneity (I^2^ = 0%, τ^2^ = 0 (0 to 3.56).

#### 3.2.5. Length of Stay

Kim et al. (2022) reported a shorter LOS with TD resection (mean difference [MD] −4 days, 95% CI −5 to −3), while Li et al. (2023) and Yoshida et al. showed no significant difference (MD 0 days, 95% CI −2 to 1, MD 3 days, 95% CI −1 to 7) [29,30]. Oshikiri et al. (2019) found longer LOS with TD resection (MD 6 days, 95% CI: 4 to 8) [13]. The pooled MD was 1 day (95% CI −6 to 8, *p* = 0.70), with high heterogeneity (I^2^ = 97%, τ^2^ = 18 (5 to >176)).

#### 3.2.6. Lymph Node Yield

LNY was higher with TD resection in most studies. Kim et al. (2022) showed no significant difference (MD 0, 95% CI −1 to 2), while Li et al. (2023) reported higher LNY with TD resection (MD 3, 95% CI: 1 to 6) [29,30]. Other studies, including Matsuda et al. (2016) and Oshikiri et al. (2022), also reported increased LNY with TD resection (MD 8, 95% CI: 5 to 10; 8, 95% CI: 4–12, respectively) [27,29,30,31,32]. The pooled MD was 4 (95% CI: 0 to 8, *p* = 0.043), which is a statistically significant difference, with heterogeneity (I^2^ = 90%, τ^2^ = 9.3 [2.4 to 90]).

### 3.3. Data Review and Meta-Analysis: Ligation vs. Preservation of the Thoracic Duct

#### 3.3.1. 5-Year Overall Survival

A total of five studies reported the 5-year OS after ligation or preservation of the TD. Hou et al. (2014) and Chen et al. (2019) reported a significantly reduced 5-year OS rate in the TD ligation group (OR: 1.22, 95% CI: 1.09 to 1.37; OR: 1.55, 95% CI: 1.26–1.91) [24,25]. The studies by Bao et al. (2020), Fei et al. (2020), and Yang et al. (2022) did not indicate a significant survival difference between the groups [33,35,37]. The pooled OR for 5-year OS was 1.15 (95% CI: 0.81–1.63, *p* = 0.33), with moderate heterogeneity (I^2^ = 76%, τ^2^ = 0.1 [0 to 0.4]).

#### 3.3.2. Chyle Leakage

**Figure 3:**
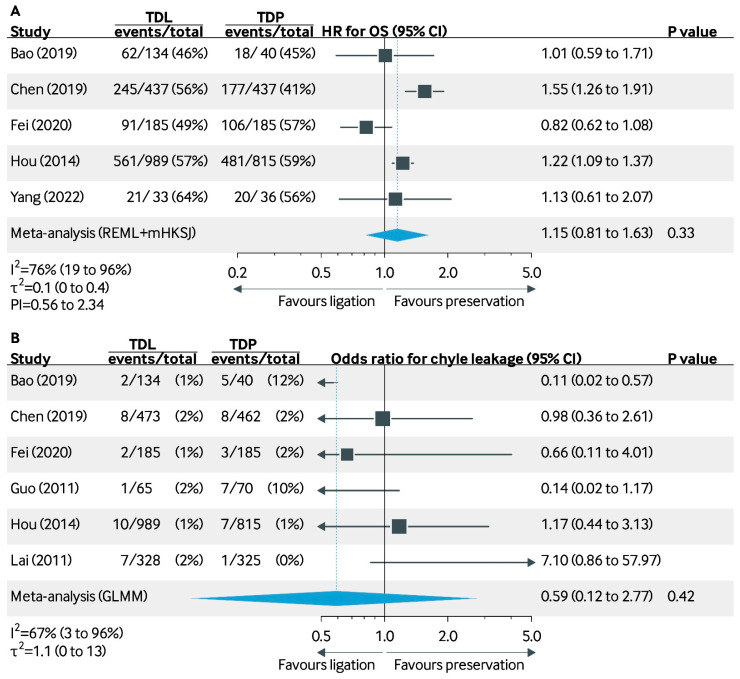
Meta-analysis of outcomes for thoracic duct ligation vs. preservation [24,25,33,34,35,36,37]. Subfigures: (**A**): 5-year Overall Survival (OS); (**B**): Incidence of Chyle Leakage. Abbreviations: TDL, thoracic duct ligation; TDP, thoracic duct preservation; REML, restricted maximum likelihood; mHKSJ, Jackson’s modification of the Hartung-Knapp-Sidik-Jonkman variance correction; GLMM, generalized linear mixed model; PI, prediction interval.

The incidence of chyle leakage in the TD ligation group ranged from 1% to 2%, whereas in the TD preservation group, this ranged from 0% to 12% (Figure 3). Bao et al. (2020) [33] reported a significantly reduced risk of chyle leakage in favor of the ligation group (OR: 0.11, 95% CI: 0.02–0.57) [30]. A total of 5 studies found no significant difference in the incidence of chyle leakage between the two groups. The pooled OR was 0.59 (95% CI: 0.12–2.77), with moderate heterogeneity (I^2^ = 67%, τ2 = 1.1 [0 to 13]) [24,25,34,35,36].

#### 3.3.3. Incidence of Postoperative Complications

Only one study reported the overall complication rate after TD ligation and preservation, showing no significant difference between the two groups (Guo et al. 2011) (OR: 0.54, 95% CI: 0.25–1.15) [34]. The complications were not classified according to the CDC. Fei et al. (2020) compared the incidence of anastomotic leakage, recurrent nerve palsy, arrhythmia, and pneumonia and found no significant differences between the ligation and preservation groups (TD ligation vs. TD preservation; 9.7% vs. 9.7%, 2.7% vs. 0.5%, 11.9% vs. 17.8%, and 18.9% vs. 22.2%, respectively) [35].

#### 3.3.4. Length of Stay

Only one study by Fei et al. (2020) reported the difference in LOS in days after TD ligation or preservation, revealing no significant difference between the two groups (MD: −0.7, 95% CI: −4.6 to 3.1) [35].

## 4. Discussion

This systematic review and meta-analysis investigated the 5-year OS, overall morbidity, incidence of chyle leakage, LOS, and LNY after esophagectomy with TD resection, TD ligation, and TD preservation. An increased incidence of chyle leakage and a higher LNY were observed after TD resection. TD ligation was similar to TD preservation across all outcome domains, including 5-year OS and morbidity. This study thereby illustrates the conflicting evidence on the impact of TD resection or TD ligation on survival.

Esophagectomy with TD resection is the standard treatment for esophageal cancer, providing accurate staging, local disease control, and possibly improved oncological outcomes. However, there remains ongoing debate regarding the ideal extent of lymph node dissection during an esophagectomy. The association between surgical radicality, as measured by LNY, and survival outcomes remains controversial, potentially due to the known variability in the extent of lymphadenectomy, anatomical boundaries, and histopathological reporting across institutions [38,39]. Nevertheless, this meta-analysis did not find evidence for an association of TD resection with improved survival.

Mediastinal lymphadenectomy based on SCC tumor location has been advocated for in the Japanese guidelines and includes the removal of periductal lymph nodes located in the fatty tissue surrounding the TD for all locations of SCC. TD resection, when performed along with dissection of periductal and para-aortic adipose tissue, allows for the removal of these lymph nodes, although dissection is also possible close to the duct while preserving it. This will probably be easier and safer under fluorescent guidance [40]. Udagawa et al. reported that metastasis to lymph nodes along the TD occurred in 2.2% of early-stage (pT1/T2) and 10% of advanced-stage (pT3/T4) SCC cases [11]. Supporting these findings, a Dutch study on AC identified metastatic lymph nodes in the periductal area in 10% of pT3 stage patients [2]. Moreover, Matsuda et al. demonstrated that lymph node metastases around the TD were found in 11% of SCC cases, with the rate rising from 4% in early-stage tumors to 26% in more advanced stages [23].

In this study, TDR was associated with similar postoperative outcomes, except for a higher risk of chyle leak (*p* = 0.044). A possible explanation is that resection of the TD may heighten the risk of iatrogenic injury and increased lymphatic pressure, exacerbating the likelihood of leakage. Managing the chyle leakage often involves a medium-chain triglycerides diet with fluid and electrolyte replacement, parenteral nutrition, thoracic duct embolization, pleurodesis, or reoperations. Therefore, chyle leakage post-esophagectomy is associated with higher morbidity, prolonged hospital stay, and thoracic drainage, and has even been related to poorer survival [10].

A key strength of this study is the use of the Hartung-Knapp-Sidik-Jonkman adjustment in our meta-analysis, as recommended by the Cochrane Handbook for Systematic Reviews of Interventions. This adjustment is especially relevant, as standard meta-analysis models can produce *p* values that are too low, with type I error rates that can exceed 20% rather than the nominal 5%. In fact, a previous study found that 25% of statistically significant findings from standard meta-analyses were non-significant when re-analyzed with the Hartung-Knapp-Sidik-Jonkman adjustment [41]. These findings are especially concerning, given the importance of meta-analyses in evidence-based guidelines and clinical decision-making. As such, the use of the Hartung-Knapp-Sidik-Jonkman adjustment in this study enhances the robustness and reliability of our findings, ensuring more accurate estimates of intervention effects. The fact that different statistical methods can lead to divergent conclusions is illustrated by our finding that contrasts with a previous review, which reported that TD ligation was associated with poorer survival—a conclusion we could not confirm with our analysis [42]. It should be noted, however, that due to the limited reporting of time-to-event data and effect measures in the original studies, some form of data modeling was required to pool the data. This has a negative impact on the certainty of evidence and underscores the necessity for studies with a higher grade of evidence and complete reporting.

This systematic review and meta-analysis has important limitations that affect the generalizability of its findings. The included studies primarily report on outcomes for TD resection in patients with SCC. The cohort with AC, more common in the West, comprises less than 1% of the total cases in this study. Consequently, the current findings remain uncertain and cannot be reliably extrapolated to patients with AC. Three studies by Oshikiri et al. were included in the 5-year survival analysis, reporting survival benefits for TD resection. This introduces significant potential bias due to the possibility of duplicate cases, which represents a major limitation of the analysis. This similarly applied to the two studies by Matsuda et al., though their impact on the results was less significant since they could not be pooled in the same meta-analyses due to differences in reported outcome domains. The majority of the studies in this review were retrospective, with only two randomized studies conducted to date, but solely comparing TD ligation with TD preservation [36,37]. Additionally, a number of studies conducted 1:1 propensity-score matching, adjusting for key clinical confounders like age and tumor stage. Studies predominantly originated in Japan and China, where neoadjuvant therapy is less commonly used, which may have introduced allocation bias. The decision between TD preservation and TD resection, potentially influenced by tumor location, T-stage, and N-stage, creates selection bias and inherently heterogeneous groups, requiring caution in drawing firm conclusions. Additionally, there was significant variation in operative approaches, likely reflecting the evolving use of minimally invasive techniques, which have gained popularity in more recent years [43].

## 5. Conclusions

In this meta-analysis, TD resection resulted in a higher LNY and an increased incidence of chyle leakage compared to TD preservation. Despite the difference in LNY, resection of the TD did not cause a difference in survival when compared to TD preservation. TD ligation does not significantly affect oncological or surgical outcomes compared to TD preservation. However, the certainty of evidence of these findings is limited due to the risk of bias in the included studies. Besides, the results of this study may be primarily applicable to patients with SCC, as it represents the majority of the cohort. Given the limited data and lack of consistent oncological benefit, routine TD resection or TD ligation cannot yet be recommended in the surgical management of this disease. These findings suggest the need for a more cautious interpretation of the existing evidence concerning the balance between benefits and potential harms of TD resection. Randomized controlled trials comparing TD resection and TD preservation are necessary to definitively resolve this ongoing debate and are currently in preparation (TUPEC-trial).

## Figures and Tables

**Table 1:** TD resection vs. preservation.

Author (Year)	Country	Study Design	Time Frame	Total Included Patients (n)	Neoadjuvant Therapy (CT or CRT)	Histology Tumor SCC/AC/Other	Risk of Bias (ROBINS-I)
Oshikiri (2022) [26]	Japan	Retrospective + PSM	2007–2012	170	170	170/0/0	Moderate
Tanaka (2021) [27]	Japan	Retrospective + PSM	2000–2017	1284	1032	1284/0/0	Moderate
Fujisawa (2021) [28]	Japan	Retrospective + PSM	2015–2019	174	98	NR	Moderate
Kim (2022) [29]	Korea	Retrospective	2013–2019	232	0	232/0/0	Moderate
Li (2023) [30]	China	Retrospective	2019–2020	200	68	200/0/0	Moderate
Yoshida (2019) [22]	Japan	Retrospective	2005–2018	537	233	NR	Serious
Oshikiri (2019) [13]	Japan	Retrospective + PSM	2010–2014	244	174	174	Serious
Oshikiri (2023) [31]	Japan	Retrospective + PSM	2007–2012	3274	1320	3214/62/0	Moderate
Matsuda (2019) [32]	Japan	Retrospective	2004–2016	235	NR	235/0/0	Moderate
Matsuda (2016) [23]	Japan	Retrospective	2004–2015	256	139	256/0/0	Serious

Abbreviations: AC, adenocarcinoma; CT, chemotherapy; CRT, chemoradiotherapy; NR, not reported; PSM, propensity score matching; SCC, squamous cell carcinoma; TD, thoracic duct.

**Table 2:** TD ligation vs. preservation.

Author (Year)	Country	Study Design	Time Frame	Total Included Patients (n)	Neoadjuvant therapy (CT or CRT)	Histology Tumor SCC/AC/Other	Risk of Bias (ROBINS-I or ROB-2 *)
Bao (2020) [33]	China	Retrospective	2009–2018	600	48	587/13/0	Moderate
Guo (2011) [34]	China	Retrospective	2009–2010	135	NR	NR	Moderate
Hou (2014) [24]	China	Retrospective	1996–2008	1793	116	1534/187/83	Serious
Chen (2019) [25]	China	Retrospective + PSM	2003–2013	874	112	874/0/0	Serious
Fei (2020) [35]	China	Retrospective + PSM	2012–2014	609	9	231/106/31	Moderate
Lai (2011) [36]	China	RCT	2004–2009	653	NR	577/65/11	Moderate *
Yang (2022) [37]	China	RCT	2016–2021	69	NR	69/0/0	Moderate*

Abbreviations: AC, adenocarcinoma; CT, chemotherapy; CRT, chemoradiotherapy; NR, not reported; PSM, propensity score matching; SCC, squamous cell carcinoma; RCT, randomized controlled trial; TD, thoracic duct. * Lai et al and Yang et al were assessed via ROB-2 instead of ROBINS-1.

## Data Availability

Data is available upon reasonable request.

## Supplementary Materials

The following supporting information can be downloaded at: https://www.mdpi.com/article/10.3390/cancers17060967/s1, File S1: PRISMA_2020_checklist_final; File S2: Search strategy; File S3: GRADE_final. References [16,44,45] are cited in the Supplementary Materials.
