# Supplementary material for: Resection vs. Ligation vs. Preservation of the Thoracic Duct During Esophagectomy for Cancer: A Systematic Review and Meta-Analysis
Source: Cancers (Basel). 2025 Mar 13;17(6):967. doi: 10.3390/cancers17060967 (PMC11940447; doi:10.3390/cancers17060967)
Supplement: Supplementary file 1 [file cancers-17-00967-s001.zip › Supplementary File S2. Search strategy.pdf]

Thoracic duct resection versus preservation in Esophageal cancer surgery

Dillen van der Aa, David Nijssen, Suzanne Gisbertz

Search: Faridi Jamaludin, Amsterdam UMC location University of Amsterdam, Medical Library AMC, Meibergdreef 9, Amsterdam, The Netherlands

UPDATE SEARCH 27-10-2023 – 3-6-2024

3-6-2024:

Databases:		
Medline (Ovid), Embase (Ovid),<br><br>Cochrane CENTRAL	Before deduplication	After deduplication*
Total	152	73

*Deduplication with: Dedupendnote.nl - Lobbestael, G. (2023). DedupEndNote (Version 1.0.0) [Computer software]. https://github.com/globbestael/DedupEndNote

MEDLINE (OVID):

UPDATE SEARCH 27-10-2023 t/m 3-6-2024

Database(s): Ovid MEDLINE(R) ALL 1946 to May 30, 2024
Search Strategy:

#	Searches	Results
1	exp Esophageal Neoplasms/	61125
2	exp Esophagus/ and exp Neoplasms/	13704
3	((esophag* or oesophag*) adj9 (cancer* or carcinom* or neoplas* or metasta* or tumo*)).ti,ab,kf.	71468
4	1 or 2 or 3	89383
5	Thoracic Duct/ or Chylothorax/ or (thoracic duct* or thoracic lymphatic duct* or thoracic lymph duct* or thorax duct* or ductus thoracicus or cisterna chyli or cisterna chylus).ti,ab,kf.	8763
6	Esophagectomy/ or Ligation/ or Constriction/ or exp surgical procedures, operative/ or surgery.fs. or (resect* or preservat* or surg* or ligation* or ligature* or constriction* or clipation* or clipated or clipping* or esophagectom* or esophagogastrectom* or oesophagectom* or oesophagogastrectom*).ti,ab,kf.	5508452
7	4 and 5 and 6	298
8	limit 7 to yr="2023 -Current"	31

EMBASE (OVID):

UPDATE SEARCH 27-10-2023 t/m 3-6-2024

Database(s): Embase Classic+Embase 1947 to 2024 May 31
Search Strategy:

#	Searches	Results
1	exp esophagus tumor/	114093
2	((esophag* or oesophag*) adj9 (cancer* or carcinom* or neoplas* or metasta* or tumo*)).ti,ab,kf.	105532
3	1 or 2	139535
4	thoracic duct/ or chylothorax/	14116
5	(thoracic duct* or thoracic lymphatic duct* or thoracic lymph duct* or thorax duct* or ductus thoracicus or cisterna chyli or cisterna chylus or chylothorax).ti,ab,kf.	12362
6	4 or 5	16420
7	exp ligation/ or exp surgery/ or surgery.fs. or exp esophagectomy/	7105463
8	(resect* or preservat* or surg* or ligation* or ligature* or constriction* or clipation* or clipated or clipping* or esophagectom* or esophagogastrectom* or oesophagectom* or oesophagogastrectom*).ti,ab,kf.	4064136
9	7 or 8	8064559
10	3 and 6 and 9	1180
11	limit 10 to yr="2023 -Current"	120

UPDATE SEARCH 27-10-2023 t/m 3-6-2024:

Cochrane Central Register of Controlled Trials

Issue 5 of 12, May 2024

ID Search Hits

#1 ((esophag* or oesophag*) near/9 (cancer* or carcinom* or neoplas* or metasta* or tumo*)):ti,ab,kw 7445

#2 (thoracic duct* or thoracic lymphatic duct* or thoracic lymph duct* or thorax duct* or ductus thoracicus or cisterna chyli or cisterna chylus or chylothorax):ti,ab,kw 285

#3 (resect* or preservat* or surg* or ligation* or ligature* or constriction* or clipation* or clipated or clipping* or esophagectom* or esophagogastrectom* or oesophagectom* or oesophagogastrectom*):ti,ab,kw
354892

#4 #1 AND #2 AND #3 with Cochrane Library publication date Between Oct 2023 and Jun 2024, in Trials
1

Additional added:

Lymphatic thoracic duct ligation modulates the serum levels of IL-1beta and IL-10 after intestinal ischemia/reperfusion in rats with the involvement of tumor necrosis factor alpha and nitric oxide.

Cavriani G, Domingos HV, Oliveira-Filho RM, Sudo-Hayashi LS, Vargaftig BB, de Lima WT

Shock. 27(2):209-13, 2007 Feb.

A review of the surgery of the thoracic duct.

ROSS JK

Thorax. 16:12-21, 1961 Mar.
