# Supplementary material for: Resection vs. Ligation vs. Preservation of the Thoracic Duct During Esophagectomy for Cancer: A Systematic Review and Meta-Analysis
Source: Cancers (Basel). 2025 Mar 13;17(6):967. doi: 10.3390/cancers17060967 (PMC11940447; doi:10.3390/cancers17060967)
Supplement: Supplementary file 1 [file cancers-17-00967-s001.zip › Supplementary File S3. GRADE_final.pdf]

Supplementary File S3

Supplementary Table S2: Certainty of evidence of thoracic duct resection (TDR) versus thoracic duct preservation (TDP) evaluated by GRADE methodology.

Certainty of evidence							No. of patients		Effect	Certainty	Importance
**No. of studies**	**Study design**	**Risk of Bias**	**Inconsistency**	**Indirectness**	**Imprecision**	**Other considerations**	TDR	TDP			
5-year overall survival											
5 studies	Observational	Serious	Serious	Serious	Serious	Propensity score matching in 4/5 studies	2628	2833	HR: 0.94 (95% CI 0.76-1.17, p = 0.48)	●○○○ Very Low	Important
Chyle leakage											
6 studies	Observational	Serious	Not serious	Not serious	Not serious	Propensity score matching in 2/6 studies	32/744	15/826	OR: 2.41 (95% CI 1.04-5.61, p = 0.044)	●●●○ Moderate	Important
Major morbidity (Clavien-Dindo ≥3)											
3 studies	Observational	Serious	Not serious	Not serious	Serious	Propensity score matching in 1/3 studies	67/341	61/570	OR: 1.45 (95% CI 0.59-3.54, p=0.22)	●○○○ Very Low	Important
Length of stay											
4 studies	Observational	Not serious	Serious	Not serious	Serious	Propensity score matching in ¼ studies	404	647	MD: 1 day (95% CI -6 to 8, p=0.70)	●○○○ Very Low	Important
Lymph node yield											
5 studies	Observational	Not serious	Serious	Not serious	Serious	Propensity score matching in 2/5 studies	1057	979	MD: 4 (95% CI 0 to 8, p=0.043)	●●○○ Low	Important

Supplementary Table S3: Certainty of evidence of thoracic duct ligation (TDL) versus thoracic duct preservation (TDP) evaluated by Grade methodology.

Certainty of evidence							No. of patients		Effect	Certainty	Importance
**No. of studies**	**Study design**	**Risk of Bias**	**Inconsistency**	**Indirectness**	**Imprecision**	**Other considerations**	TDL	TDP			
5-year overall survival											
5 studies	Observational + randomized	Serious	Not serious	Serious	Serious	Strong association	1778	1513	HR: 1.15 (95% CI 0.81 to 1.63, p=0.33)	●●◯◯ Low	Important
Chyle leakage											
6 studies	Observational + randomized	Not serious	Serious	Not serious	Not serious	Confounding would reduce effect	30/2174	31/1897	OR: 0.59 (95% CI 0.12 to 2.77, p=0.42)	●●◯◯ Low	Important
